# Experiences of Intimate Partner Violence during Lockdown and the COVID-19 Pandemic

**DOI:** 10.1007/s10896-021-00260-x

**Published:** 2021-02-26

**Authors:** Minna Lyons, Gayle Brewer

**Affiliations:** Department of Psychology, University of Liverpool, Liverpool, L69 7ZA UK

**Keywords:** COVID-19, Domestic violence, Intimate partner violence, Online forum, Partner abuse, Pandemic

## Abstract

Previous studies have demonstrated that there is an increase in Intimate Partner Violence (IPV) during times of crisis (e.g., financial, environmental, or socio-political situations). The COVID-19 pandemic has triggered an unprecedented global health and financial tragedy, but research is yet to establish exactly how the situation may impact on IPV. The present study investigates victims’ experience of IPV during lockdown and the COVID-19 pandemic. We report a qualitative thematic analysis of 50 discussion forum posts written by victims of IPV. Of these, 48 forum posts were written by female victims of male perpetrated violence. All forum posts were obtained from the popular online platform, Reddit. We identified four themes associated with IPV victims’ experiences during lockdown and the global pandemic: (i) Use of COVID-19 by the Abuser, (ii) Service Disruption, (iii) Preparation to Leave, and (iv) Factors Increasing Abuse or Distress. The COVID-19 pandemic has had a substantial impact on those living with IPV, often increasing the severity of IPV experienced. The experiences of those affected by IPV during this period inform interventions and the guidance and support provided to IPV victims during times of crisis.

The outbreak of the coronavirus (COVID-19) pandemic is likely to have severe negative consequences for victims of intimate partner violence (IPV; Boserup et al. 2020; Bradbury-Jones and Isham 2020; Peterman et al. 2020; Usher et al. 2020). IPV consists of a wide range of behaviours between current or former romantic partners, encompassing sexual, psychological, physical, and financial abuse of differing degrees (Peterman et al. 2020). Although male perpetrator-female victim is the most common pattern, female perpetrators and male victims are not unusual (Hines and Douglas 2009). In addition, IPV occurs in same-sex relationships at a prevalence comparable to heterosexual relationships (Rollè et al. 2018). Research has linked times of uncertainty (e.g., natural disasters, civil unrest, virus outbreaks, economic insecurity) to increased violence within families, including abuse directed towards romantic partners (see Peterman et al. 2020 for a review). Indeed, there are already anecdotal accounts reporting a pandemic-related escalation of violence against women and girls in several regions of the world (Peterman et al. 2020). In order to develop effective strategies for intervention and prevention, it is essential to gain knowledge of the dynamics that underlie the exacerbated incidences of partner violence during crises such as the COVID-19 pandemic.

There are several potential direct and indirect mechanisms influencing the increase of IPV perpetration during the virus outbreak (Peterman et al. 2020). First, the pandemic has increased rates of unemployment to unprecedented levels (Kawohl and Nordt 2020), pushing many households into poverty. IPV has well-recorded links with financial stressors (e.g., Lucero et al. 2016; Schwab-Reese et al. 2016), and could have complicated interactions with factors such as emasculation and alcohol use (Peralta et al. 2010). In addition, financial hardship may result in a reduced likelihood of the victim leaving the abuser. Financial abuse could, in fact, be one of the many strategies for the perpetrators to prevent their victim from escaping (Eriksson and Ulmestig 2017). In effect, financial hardship can increase stress and put more strain on relationships, as well as reduce opportunities for the victim to leave.

Second, social isolation measures related to the pandemic leave many victims without social contacts, housebound with the perpetrator. Social isolation has been associated with increased risk of IPV in some contexts (e.g., in migrant women; Kim 2019; in rural areas; Lanier and Maume 2009), and potentially prevents the victim from seeking help from others (van Gelder et al. 2020). Indeed, isolating victims from their social support network is a common strategy employed by perpetrators to control their victims. Thus, increased contact with the perpetrator, coupled with reduced social contacts with others, are likely to put many already vulnerable victims into even more precarious situations.

Third, the services that might normally be available to IPV victims may simply not be there, or function at a reduced capacity during a pandemic. Health care providers and emergency personnel are often the first point of contact for IPV victims, and play a major role in screening for IPV, identifying it, and encouraging disclosure (Bradley et al. 2020). Because of limited access to health care during the pandemic, many incidences of IPV may not be identified. In addition, police intervention is often the first response to IPV (Cheng and Lo 2019). Due to social restrictions and police engagement in new roles such as coordination and enforcement of local lockdowns, victims and bystanders (e.g., neighbours) may be less likely to contact the police during incidence of physical violence. It is possible that the pandemic leaves many victims unnoticed, without the help of authorities and health care providers.

Fourth, the pandemic may be a tool for the abusers to exert their power further. Some of the well-recorded IPV tactics include coercive control, which has been defined as “a strategic course of oppressive conduct that is typically characterized by frequent, but low-level physical abuse and sexual coercion in combination with tactics to intimidate, degrade, isolate, and control victims” (Stark 2013, p. 18). The abusers may use the social isolation measures during the pandemic as an excuse for further controlling and isolating the victims. Coercive control has been identified as one of the important predictors of femicide (Campbell et al. 2003), highlighting the concern about murder victims as “collateral damage” of the pandemic. The pandemic could even escalate the journey of the perpetrator from coercive control to murder (see Monckton Smith 2019).

The aim of the present study is to qualitatively understand the experiences of IPV victims during the pandemic. We are utilising online discussion forums, a data source that has become a popular tool in both qualitative (e.g., Newberry 2017) and quantitative (e.g., Lyons et al. 2018; Lyons et al. 2020) psychological research. Online communities can provide a constructive forum for advice, support, and social contact in those who experience IPV (Hurley et al. 2007; Lindgren 2014; Newberry 2017). IPV victimisation is often related to shame, self-blame, and social stigma (Eckstein 2016; Lim et al. 2015), which may make it harder in terms of seeking support face-to-face (Overstreet and Quinn 2013). The online environment facilitates interacting and sharing stories with others using an anonymous username, reducing stigma relating to disclosure and providing a degree of safeguarding. The personal stories of IPV victims will be important for understanding the complicated issues that the global health crisis may impose on vulnerable people.

## Method

### Selection of Forum Posts

In this research, we utilised the highly popular social networking discussion forum platform, Reddit. This platform has more than 10,000 user-generated “subreddits”, online communities that are unified by common interests (Widman 2020). The veil of anonymity and shared experiences make it easier for the users to openly talk about stigmatising issues that may be more difficult to discuss face-to-face. Hence Reddit has been successfully used to research sensitive topics such as pro-eating disorders (Sowles et al. 2018), incel communities (Maxwell et al. 2020), and mental illness (De Choudhury and De 2014). Reddit has also been used by victims of IPV and sexual violence as a supportive environment in which experiences can be shared and acknowledged (O’Neill 2018; Schrading et al. 2015). Indeed, the use of these online communities may be empowering and reduce social isolation (van Uden-Kraan et al. 2009). Therefore, Reddit has the potential to provide an insight into user experiences in a manner unprompted by researcher priorities or assumptions.

For the present study, we searched Reddit for IPV related subreddits by using the search words “domestic violence, domestic abuse, intimate partner violence, abusive relationships, partner abuse”. We identified three relevant sites, each with a large number of users (at the point of data collection, 10,100, 12,300, and 27,100). We selected posts that were submitted between 1st March and 10th May 2020, a period in which a substantial proportion of the global population had experienced or were anticipating lockdown.

Upon entering each subreddit site, we searched for relevant posts using the words “COVID, corona, virus, and pandemic”. We went through the list of threads under each search word, selecting posts (and responses to the posts) that filled the inclusion criteria. The inclusion criteria were the following: (i) the posts had to discuss PERSONAL experiences during the pandemic. Posts that were discussing the experiences of someone else, giving advice without sharing their experiences, or did not mention COVID-19 at all were excluded. (ii) the posts had to discuss experiences of IPV victimisation (not other types of domestic violence or perpetration of IPV), with the abuse present prior to the pandemic.

We recorded posts by the usernames, analysing each username as one unit. We also searched for other posts by the username by clicking their name. If the person had written about IPV experiences during COVID-19 in other subreddits, those posts were also collected. The username, link to the post, perpetrator-victim relationship (i.e., male-female, female-male, male-male, female-female), and country of origin of the posts (wherever this was possible) were recorded.

### Ethical Issues

As the posts in the subreddits were publicly available, our Institutional Review Board for research involving human participants, did not require formal review and approval. However, when designing and conducting the study and reporting our findings we consulted relevant ethical guidelines, previously published discussion forum research, and available guides to discussion forum research (e.g., Smedley and Coulson 2021). In particular, we considered the public or private nature of the information shared, the potential for benefit or harm, and the feasibility of seeking informed consent when determining the appropriateness of the research (Eysenbach and Till 2001; Roberts 2015).

We analysed posts available to the general public without registration or log in and adopted a number of measures in accordance with professional body guidelines (e.g., British Psychological Society 2017) in order to protect the anonymity of the forum users. We are not revealing their online usernames, have slightly altered the wording of the quotes in this report, and include brief quotations rather than lengthy forum posts. To further address this issue, we entered each quote into both Google (the most widely used search engine) and Reddit (the discussion forum platform used to obtain posts), and this did not lead to the original posts. We are not reporting the name of the subreddits used in the study either. Altogether, we collected 50 posts written by victims of IPV on the forums identified as relevant for the topic. We finished data collection after all the relevant posts were found. Most (48) were female victims reporting abuse from a male perpetrator. Although in most cases, it was not possible to trace the country of origin, 22 posts were from the US, two from the UK, one from Canada, one from Australia, and one from Cambodia.

### Data Analysis

Two researchers independently analysed the datafile using inductive thematic analysis (Braun and Clarke 2006). This analytical method has been used previously in the context of discussion forum posts on IPV (e.g., Newberry 2017), and was deemed as the most appropriate for understanding personal stories within the context of the pandemic. The researchers read the forum posts several times, and established initial codes independent from each other, utilising separate word files as an audit trail. The researchers then discussed the codes, removed any duplicates, amalgamated similar codes, and investigated any discrepancies between the coders.

After agreement on the coding system, we then organised the codes into broader themes in order to establish a preliminary thematic framework. For example, the limited shelter access, contacting law enforcement, and disruption to counselling codes later contributed to the Service Disruption theme. This was done by carefully examining any similarities and differences between codes and critically appraising the relationships between the codes. The final themes were agreed by both researchers after checking analytical interpretations for any discrepancies and it was clear that data saturation had been reached after analysis of the 50 discussion forum posts. We applied Leininger’s (1994) six criteria (credibility, confirmability, meaning in context, recurrent patterning, saturation, and transferability) when assessing the trustworthiness of our findings. These criteria are specifically intended for use with qualitative data and are consistent with the assumptions and goals of the qualitative paradigm.

Credibility. The researchers discussed their interpretations of the findings extensively, acknowledging their potential biases, and trying to adopt the perspective of the informants. We recognise that credibility is somewhat limited by the lack of participant involvement in the interpretation of the findings. However, we note that the data were posts created by users, unprompted by researcher priorities or assumptions and therefore may have greater credibility than other approaches. Confirmability. At times, it would have been beneficial to obtain clarification for some of the posts, which was not possible due to the nature of the study. However, the discussion forum posts are thought to be true, honest reflection of personal experience. Meaning-in-context. We recognize that the interpretations of the data are compatible only within the specific context addressed (i.e., the COVID-19 pandemic.

Recurrent Patterning. The texts within the posts were often in similar sequences, telling similar kind of stories (e.g., pandemic stressors increasing abuse, with victims preparing to leave). We felt that the data did include recurring experiences across multiple posters. Saturation. Although other similar studies have used larger number of posts (e.g., O’Neill 2018), our data collection was limited by the number of available posts that fit the exclusion criteria. However, both researchers agreed that when reaching the 50 posts, data saturation was reached, and no new codes/themes were emerging at this point. Transferability. The themes can be partially transferred to reflect the experiences of people in other circumstances in the pandemic. However, it is important to note that the aim of our study is not to produce findings that may be generalized to other (i.e., non-pandemic) contexts, it is to understand the experiences of those affected by IPV during the pandemic in order to inform interventions and the guidance and support provided to IPV victims.

## Results

We identified a number of themes relating to experiences of IPV during the COVID-19 pandemic. Forum posts typically also contained non-COVID-19 information (e.g., describing the abuse, providing relationship history). These themes are not reported here if they did not directly relate to experiencing IPV in the context of the pandemic. However, it is worth noting that the posts described a deep history of IPV prior to the pandemic. Therefore, the posts relate to a continuation or escalation (none described a reduction) of abuse rather than abuse initiated after the start of the pandemic and findings cannot inform our understanding of the experiences of those who first became victims of IPV during the pandemic. The four themes extracted from the data were (i) Use of COVID-19 by the Abuser, (ii) Service Disruption, (iii) Preparation to Leave, and (iv) Factors Increasing Abuse or Distress.

### Use of COVID-19 by the Abuser

Perpetrators frequently capitalized on the pandemic and incorporated it into the abuse. For example, “He’s using it as an excuse to try to throw me out of the house” (female victim, country not disclosed). Abusers also threatened victims or punished ‘unacceptable’ behaviour during the pandemic such as leaving the house during lockdown. As described on one forum post “She says she will “kill me” for putting people at risk” (male victim, U.S.A.). Similarly, another woman stated “He yelled at me after I went for a walk, he says I am selfish and “retarded”, he’s doing it to try to control me” (female victim, country not disclosed). In some instances, perpetrators made false claims to control a partner’s movements, such as “He called the airline and said I had tested positive so that they wouldn’t let me on the plane” (female victim, U.S.A.). Some posts also described perpetrators threatening to purchase a weapon, both in countries where gun ownership is legal and illegal. Potential social unrest during the pandemic was often provided as the reason for acquiring the weapon. For example, “He says he is going to buy a gun as people go crazy during the pandemic and might rob him…he is using it as an excuse to get a gun” (female victim, Australia).

### Service Disruption

COVID-19 caused considerable disruption to available services, including specialist domestic violence services (such as shelters) and associated support (e.g., counselling). Forum posts often commented that “The DV shelters are all full!” (female victim, country not disclosed), “Shelters are at capacity…everything is in lockdown because of COVID” (female victim, U.K.), and “The shelter won’t take or release people during COVID” (female victim, U.S.A). Support services often became unavailable, exacerbating the impact of the IPV. For example, “He was seeing a counsellor to help him with his anger but he had to stop due to COVID-19” (female victim, country not disclosed) and “I normally see an onsite therapist at work without him knowing but I can’t do that now” (female victim, country not disclosed). Disruption to legal proceedings often increased anxiety and the risks posed to IPV victims. For example, “My abuser is going to be released from jail because prisoners and guards have tested positive… I’m shocked and very scared… I worry for my safety and the safety of my children” (female victim, U.K.). Similarly, court cases were delayed “The court case is pending but I don’t know when it will happen now because of COVID” (female victim, U.S.A.). Regular services that support victims leaving their abuser (e.g., transportation) have also been affected. As stated by one woman, “There are hardly any flights and then I would have to quarantine for two weeks” (female victim, Cambodia).

### Preparation to Leave

Many victims reported that they were ready to leave their abusive partner and that the pandemic had interrupted their attempt to leave. For example, “I should have moved by now…The pandemic put everything on hold” (female victim, Australia) and “I was hoping to leave before the pandemic hit, now I am stuck here” (female victim, U.S.A.). Other individuals were using the lockdown to prepare to leave. For example, “I have been contacting houses to move into” (female victim, Australia) and “I’m using time to make an escape plan, I’m trying to find a way out” (female victim, U.S.A.). One victim explained “I’m using quarantine to make a plan to get out of this situation. I’ve made a secret email and packed a bag” (female victim, U.S.A.). Financial resources were particularly important. For example, victims reported “I am waiting for the payment, so I can grab the kids and leave” (female victim, U.S.A.). Highlighting the importance of the approach adopted by each country one victim stated “I need money to escape…In Australia we receive a payment due to COVID…I’m using this to escape him…As soon as I get a payment I’m using it for a deposit and leaving” (female victim, Australia).

### Factors Increasing Abuse or Distress

A range of factors increased the prevalence and intensity of the abuse or the victim’s IPV related distress. These could be identified as financial stress, increased time together, increased alcohol/drug use, pre-existing health issues of the victim or the abuser, and the presence of vulnerable others (e.g., children or pets). For example, alcohol use featured in many posts, “It gets worse when he drinks and he does this a lot” (female victim, Canada). Those living with their abuser (either permanently or because they self-isolated together on a temporary basis) were particularly distressed. In some cases, there was evidence of coercive control by the abuser, for example, “I can’t cope living with a monster anymore, everything is controlled by him. I can’t be confined to a house all day with my abuser” (female victim, Australia). The increased time spent together because of quarantine and social isolation rules seemed to be particularly challenging for many, resulting in the victim feeling like a prisoner, “I’m stuck with him in a house now, can’t do anything, and feeling paralyzed. In the past few weeks I have had suicidal thoughts every day” (female victim, country not disclosed). Similarly, another stated “Quarantine sucks, no escape from each other when we are upset. He gaslights me all the time… I don’t know where to go from here” (female victim, country not disclosed). The presence of vulnerable others also added to the victims’ distress. Many of the victims were quarantined in the house with children or pets. For example, one female victim voiced her concerns over harming her child saying “Being stuck with him is so hard. He hasn’t hurt our child before but I am afraid it might happen soon. I’ve been trying to keep my son out of harms way” (female victim, U.S.A.).

They often displayed desperation “I’m going to end up killing myself during isolation…I can’t deal with it anymore. How do I avoid him in the same house” (female victim, Australia). Financial issues (including job loss) were also important. These included the perpetrator having fewer financial resources and becoming more stressed, unpredictable, and abusive and the victim having fewer financial resources and therefore becoming more financially dependent on the abusive partner. For example, “I can’t afford to move out because there is less work” (female victim, Cambodia), “He has been out of work and his behaviour has escalated” (female victim, U.S.A.), and “I lost my job due to COVID. I’m living off his income and unemployment” (female victim, country not disclosed). Distress was exacerbated by isolation from the wider social support network though people were often worried that visiting family or friends could increase the risk of COVID-19. For example, “My mom and dad are at risk and live with someone with cancer. I wouldn’t want to risk their lives” (female victim, U.S.A).

## Discussion

The current study investigated victims’ experiences of IPV during the COVID-19 pandemic. The qualitative data gathered from Reddit discussion forums indicate that in relationships where there is a history of abuse, IPV has often been exacerbated by stressors related to the pandemic. No victims reported that the frequency or severity of abuse had declined during this period. Many of the concerns identified by the victims in our sample related to the issues that Peterman et al. (2020) raised in relation to IPV during the pandemic (e.g., economic uncertainty, quarantine and social isolation, reduced support service availability, inability to escape the abuse, and virus-specific sources of violence). Below, we will discuss each of the four themes extracted from our data.

### Use of COVID-19 by the Abuser

Many of the victims described how perpetrators were using the pandemic as an excuse for escalating abuse, especially increased surveillance of their partner and coercive control. Lockdown and quarantine rules typically resulted in abusers spending more time with the victim and increased opportunities for monitoring and control of their behaviour. For example, where lockdown required victims to work from home, abusers were able to observe interactions with colleagues. The increase in coercive control is particularly worrying as this has been identified as one of the risk factors for serious abuse, including femicide (Myhill and Hohl 2019). It is, therefore, important that victims of IPV are supported to recognise and respond to different forms of partner violence and indicators that the abuse is escalating. Abusers also used the pandemic to restrict their partner’s movements and contact with the outside world. Social isolation escalates the risk of violence and contributes to victim distress (Jose and Novaco 2016). Where restricted travel and social distancing regulations are introduced, additional measures are required to reduce isolation in order to lower the risk of family violence (Campbell 2020). Further, it is essential that local and national policy restricting travel or introducing social distancing also communicates exceptions to these rules, such as when a victim moves from one household to another to protect their safety.

Some of the victims were concerned about their abuser’s intentions to purchase a weapon, with abusers typically adopting the need for self-protection during the pandemic as an excuse for gun ownership. Indeed, gun ownership has increased in the U.S.A. since the start of the pandemic and gun related injuries or fatalities have increased in many regions (Hatchimonji et al. 2020; Sutherland et al. 2020). The ownership of a weapon has been identified as an important fatality risk indicator by female victims (Johnson et al. 2020), suggesting that many abuse victims are, for good reasons, fearing for their lives during the pandemic. Of course, the presence of a weapon not only impacts on the likelihood of homicide; it may also impact on the abuser’s ability to control their victim, victim distress and anxiety, and suicide rates (Lynch and Logan 2018; Mannix et al. 2020; Sorenson and Schut 2018). Therefore, law enforcement and those regulating weapon ownership must acknowledge and address the increased risk to IPV victims during times of crisis.

### Service Disruption

This theme centred on the disruption of services available to victims (e.g., shelters) and perpetrators (e.g., counselling). Victims who seek but do not receive external support are less likely to leave their abusive partner (Koepsell et al. 2006). Hence, pandemic-related disruptions to support services place the victims of IPV in a precarious position, reducing practical support and preventing the escape to shelters and/or family that live further away. Those supporting victims of IPV must ensure that support services are available remotely (e.g., online) and consider how this support can be safely accessed when victims are in quarantine with their abuser. Additional facilities (e.g., shelter accommodation) are also required to address demand during the COVID-19 pandemic (Ndedi 2020) and public pressure may be required to ensure that these facilities are available. Furthermore, termination of help (e.g., counselling) to the abuser may decrease their ability to cope with the pandemic-related stressors, leading to escalated IPV. Remote counselling available to perpetrators and victims would, therefore, also be beneficial (Mazza et al. 2020).

Services that are not primarily targeted at IPV victims or perpetrators have also been disrupted. For example, during lockdown, victims may have reduced contact with health care providers or law enforcement who often encourage victims to leave abusive partners (Morse et al. 2012) and it is important that opportunities to report partner abuse or seek advice are maintained. Alternative forms of reporting may be introduced, but the availability of these must be widely disseminated to victims through large-scale national campaigns. In the present study, victims also reported disruption to court activities and early release of prisoners due to COVID-19; this is particularly concerning as delayed prosecution and early release increase the risk of abuse from the violent partner. Further, in addition to their primary safeguarding function, court offers important opportunities to support the well-being of partner violence victims (Cerulli et al. 2011). It is, therefore, essential that disruption to judicial activities resulting in delayed prosecution, early offender release, or reduced victim support, is combined with measures that address the impact of this disruption on victims (i.e., increased risk and distress).

### Preparation to Leave

For some victims, COVID-19 (and associated lockdown measures) interrupted plans to leave their abusive partner. Others reflected on their relationship during this time (or perhaps were aware of the escalation of abuse during the pandemic) and decided to leave when possible. It is difficult to determine why some victims had reached the decision to leave prior to the pandemic and others were prompted to leave by COVID-19 and further research in this area is required. It is likely, however, that a range of individual, relational, and situational factors impacted on this process. Victims who used the pandemic period to prepare for their escape, engaged in a range of preparatory activities such as creating secure emails, organising belongings, and locating alternative accommodation. These activities encouraged hope that it would be possible to safely leave the relationship and optimism about the future. Indeed, victims are more likely to terminate an abusive relationship if they believe they have a degree of control over this (Byrne and Arias 2004).

These plans are particularly important where access to formal (e.g., shelters) and informal (e.g., family) support is limited. There is, however, little information available to victims relating to how to prepare to safely leave an abusive relationship (e.g., locating proof of identity which may be required to obtain benefits) and additional guidance should be provided. Online resources (e.g., those available on discussion forums used by IPV victims) may be particularly beneficial. Of course, for many victims, lockdown with their abuser makes such planning difficult or increases the risk of detection and victim safeguarding remains the priority. It is important to note that those who had access to increased funds as a consequence of the pandemic (i.e., Government funding) were particularly positive about their ability to leave the abusive partner. It is, therefore, essential that IPV victims are provided with the resources (including financial resources) necessary to leave the abusive partner. Greater public recognition of this issue may be required to ensure that funding is in place to support victims.

### Factors Increasing Abuse or Distress

Forum posts identified a range of factors that increased the intensity and prevalence of the IPV or exacerbated victim distress. In particular, financial pressures that increased perpetrator stress (and abusive or controlling behaviour) or victim dependence on the abusive partner were commonly discussed. The COVID-19 pandemic has had an extensive impact on the global economy (e.g., wide-scale redundancy) with many countries likely to enter recession (Coibion et al. 2020; Fernandes 2020). It is, therefore, important to recognise the consequences of the pandemic related economic crisis for wider societal issues. Of course, the impact of financial resource availability extends beyond the incidence of IPV and the victim’s ability to leave. For example, for those who have left their abusive partner, the availability of resources also has an important impact on subsequent health (Ford-Gilboe et al. 2009) suggesting longer-term consequences of these economic issues.

Other issues believed to increase the abuse or victim distress included the use of alcohol and drugs. The relationship between substance use and IPV is well-established (e.g., Caetano et al. 2001) and there are concerns that alcohol use has increased during lockdown (Clay and Parker 2020). Hence, public health measures to reduce substance use as a coping mechanism during pandemics or other national crises may help to lower IPV levels. Victims isolating with their abuser are at particular risk during the pandemic (Klostermann et al. 2020) and those with vulnerable others present in the household may be particularly concerned. Indeed, the presence of vulnerable others such as children or pets appears to influence both the incidence of abuse and the decision to enter a shelter (Hardesty et al. 2013). During the pandemic, children are at increased risk of exposure to violence or becoming victims themselves (Humphreys et al. 2020). It is important therefore, to ensure children exposed to violence during COVID-19 are supported (Ragavan et al. 2020). This may incorporate a range of measures including access to counselling and school based support.

### Limitations

The present study investigated the experiences of IPV victims during lockdown and the COVID-19 pandemic, using online forum discussion posts. Though these provide an important insight into those issues of most concern to victims, unprompted by the priorities or assumptions of the researchers, it is not possible to determine whether these posts are representative of IPV victim experiences. For example, Reddit users tend to be younger and more educated than the general population (Pew Research Center 2016). In addition, though we made note of the country of origin where listed, we have little demographic detail for the individuals posting online. In both research and practice there has little consideration of IPV experiences in racial and ethnic minorities (Lee et al. 2002). Therefore, whilst it is particularly important to investigate the experiences from those in minority groups as ethnicity may be related to COVID-19 incidence or outcomes (Pareek et al. 2020), our research cannot inform this issue. Finally, the present study did not specifically target the female victims of male perpetrators. The majority of the posts selected were, however, written by the female victims of male perpetrators. It is important to recognise that those in same-sex relationships are also at risk of IPV (Messinger 2011) as are men in relationships with female perpetrators (Carmo et al. 2011). Future studies should specifically consider these groups.

### Impact and Conclusions

The present research represents one of the first studies to gain knowledge of the dynamics that influence the increased incidence of partner violence during crises such as the COVID-19 pandemic. We investigated experiences of IPV via a qualitative thematic analysis of Reddit discussion forum posts, an approach which provides an insight into user experiences unprompted by researcher priorities or assumptions. Four themes emerged. These were Use of COVID-19 by the Abuser, Service Disruption, Preparation to Leave, and Factors Increasing Abuse or Distress. These findings inform interventions and the guidance provided to those affected by IPV. In particular, we advocate supporting victims to recognise and respond to different forms of partner violence and indicators that the abuse is escalating, and ensuring that victims are aware of exceptions to social distancing policy that allows movement to protect personal safety. Large scale national campaigns to disseminate this information and information advising victims how to safely leave an abusive relationship are recommended. It is also essential that law enforcement are aware of the increased risk to IPV victims during times of crisis and that specialist services (e.g., counselling and shelters) are protected.
